# Amelioration of Hypertension by Oryeongsan through Improvements of Body Fluid and Sodium Balance: Roles of the Renin-Angiotensin System and Atrial Natriuretic Peptide System

**DOI:** 10.1155/2022/9159292

**Published:** 2022-06-08

**Authors:** You Mee Ahn, Hye Yoom Kim, Jung Joo Yoon, Hyun Ju Kim, Yun Jung Lee, Young Gab Yun, Hyeun Kyoo Shin, Kyung Woo Cho, Dae Gill Kang, Ho Sub Lee

**Affiliations:** ^1^Hanbang Cardio-Renal Syndrome Research Center, Wonkwang University, Iksan, Republic of Korea; ^2^Herbal Medicine Research Division, Korea Institute of Oriental Medicine, 1672 Yuseong-daero, Yuseong-gu, Daejeon 34054, Republic of Korea; ^3^College of Oriental Medicine and Professional Graduate School of Oriental Medicine, Wonkwang University, Jeollabuk-do, Iksan 54538, Republic of Korea

## Abstract

Oryeongsan (Wulingsan in China and Goreisan in Japan), a formula composed of five herbal medicines, has long been used for the treatment of imbalance of the body fluid homeostasis in Asian countries. However, the mechanism by which Oryeongsan (ORS) improves the impaired body fluid and salt metabolism is not clearly defined. The present study was performed to define the role of the cardiorenal humoral system in the ORS-induced changes in blood pressure and renal function in hypertension. Experiments were performed in normotensive and two-kidney, one-clip hypertensive rats. Changes in the fluid and salt balance were measured in rats individually housed in metabolic cages. Changes in the systemic and local renin-angiotensin system (RAS) and cardiac natriuretic peptide hormone system (NPS) were evaluated. ORS water extract was administered by oral gavage (100 mg/kg daily) for 3 weeks. ORS induced diuresis and natriuresis along with an increase in glomerular filtration rate and downregulation of the Na^+^/H^+^ exchanger 3 (NHE3) and aquaporin 2 expression in the renal cortex and medulla, respectively. Furthermore, treatment with ORS significantly decreased systolic blood pressure with contraction of body sodium and water accumulation in hypertensive rats. ORS-induced changes were accompanied by modulation of the RAS and NPS, downregulation of the systemic RAS and cardiorenal expression of angiotensin-converting enzyme (ACE) and angiotensin II subtype 1 (AT_1_) receptor, and upregulation of the plasma ANP concentration and cardiorenal expression of ANP, ACE2, Mas receptor, and AT_2_ receptor. These findings indicate that ORS induces beneficial effects on the high blood pressure through modulation of the RAS and NPS of the cardiorenal system, suppression of the prohypertensive ACE-AT_1_ receptor pathway and NHE3, accentuation of the antihypertensive ACE2-Mas axis/AT_2_ receptor pathway in the kidney, suppression of the systemic RAS, and elevation of the plasma ANP levels and its synthesis in the heart. The present study provides a biological basis for the use of ORS in the treatment of impaired volume and pressure homeostasis.

## 1. Introduction

Oryeongsan (ORS, Wulingsan in China and Goreisan in Japan), a traditional oriental medicine, has long been used for the treatment of imbalance of the body fluid homeostasis. ORS, an old formula, was first mentioned in the traditional Chinese medicine book Shanghan Lun (Treatise on Febrile Diseases) written by Zhang ZhongJing in the third century. The formula is composed of five medicinal herbs, *Alisma orientale* Juzepzuk, *Poria cocos* Wolf, *Atractylodes macrocephala* Koidzumi, *Polyporus umbellatus* Fries, and *Cinnamomum cassia* Presl [1, 2]. However, the nature and precise mechanisms by which ORS improves cardiorenal function remain to be defined.

Previously, it was shown that ORS elicits diuresis and natriuresis and an increase in glomerular filtration rate (GFR) in experimental animals [2, 3]. Earlier, Lee reported on the diuretic and natriuretic actions of ORS [3]. In anesthetized rabbits, slowly injected ORS increased urine volume for about 90 min with urinary excretion of Na^+^ and Cl^−^ for about 60 min. The diuretic and natriuretic effects were accompanied by an increase in clearance for creatinine (GFR). Furthermore, the data showed that the fractional excretion of Na^+^ was not changed by ORS. From these data, Lee suggested that although renal tubular effects may not be excluded, the action mechanism of the ORS may be closely associated with an increase in GFR.

These findings were confirmed later by Ahn et al., and a part of the mechanisms involved was defined [2]. Subacute treatment with ORS increased urine volume, urinary excretion of Na^+^, and creatinine. Urinary excretion of Na^+^ was a function of the urinary excretion of creatinine which suggested the importance of the role of GFR in the natriuretic effect of ORS. Furthermore, ORS decreased urinary osmolality and Na^+^ balance. All these findings were accompanied by a decrease in plasma levels of renin activity and aldosterone. Recently, it was shown that ORS attenuates the hypertonicity-induced increase in AQP2 protein expression and its insertion into the plasma membrane of the inner medullary collecting duct cells [4]. This indicates that ORS may affect urinary concentration ability through tubular water channel proteins in the kidney. Furthermore, water extract of components of ORS, *Atractylodes macrocephala* Koidzumi [5] and *Poria cocos* Wolf [6], has similar effects on the inner medullary collecting duct cells. Oral intake of water extract of *Polyporus umbellatus* Fries, another component of herbal medicine of ORS, has also been known to inhibit AQP2 gene expression in renal medulla and increase urine volume in rats [7].

It was also shown that chronic treatment with ORS decreased high blood pressure in renovascular (two-kidney, one-clip (2K1C)) hypertensive and spontaneously hypertensive rats (SHR) [8, 9] with a decrease in plasma levels of renin and aldosterone, suppression of the angiotensin II subtype 1 (AT_1_) receptor mRNA expression, and accentuation of the ACE2 mRNA expression in the ventricular myocardium from SHR [9]. These reports suggest that ORS improves the function of the cardiorenal system. Both the RAS and cardiac natriuretic peptide hormone system (NPS) are involved in the regulation of blood pressure balance and body fluid homeostasis. The RAS, intrarenal RAS in particular, is known to be involved in the regulation of blood pressure balance and body fluid homeostasis [10–12]. The roles of the AT_1_ receptor and Na ^+^ -H^+^-exchanger isoform 3 (NHE3) of the renal proximal tubules are critical in the regulation of the blood pressure balance [13–15]. Both the RAS and NPS are involved in the regulation of glomerular afferent and efferent arteriolar function [16, 17]. Furthermore, the RAS is negatively regulated by the cardiac natriuretic peptide hormones [18, 19]. The NPS is a cross-communicating partner of the RAS in the regulation of the cardiovascular system. The purpose of the present study was to identify the effects of ORS on the regulation of renal function and blood pressure in relation to the cardiorenal humoral system in a Goldblatt model of 2K1C hypertensive rats in which the RAS is accentuated.

## 2. Materials and Methods

### 2.1. Preparation of ORS Water Extract

ORS formula is composed of five medicinal herbs: *Alisma orientale* Juzepzuk (9.375 g), *Poria cocos* Wolf (5.625 g), *Atractylodes macrocephala* Koidzumi (5.625 g), *Polyporus umbellatus* Fries (5.625 g), and *Cinnamomum cassia* Presl (1.875 g) to be 28.125 g in total for a single dose in an adult subject. The ratio of the component herbs of the herbal formula is 5 : 3:3 : 3:1 for the above herbs, respectively. Parts used of each of the herbs are root for all except cortex for *Cinnamomum* cassia Presl. The ORS formula used in the present study is the same as that of the previous reports [2, 20, 21]. The five herbs of ORS formula were purchased from Kwangmyungdang Medicinal Herbs Co., Ltd. (Ulsan, Korea). The herbs were identified by a pharmacognosist, Professor Je-Hyun Lee, Dongguk University, Gyeongju, Korea. A voucher specimen (2008-KE17-1∼KE17-5) was deposited at the Herbal Medicine Research Division, Korea Institute of Oriental Medicine. To obtain the ORS water decoction, the above five medicinal herbs were mixed (total weight 3.0 kg, about 106.7 times of composition of a single dose) and extracted in distilled water at 100°C for 2 h using an electric extractor (COSMOS-660, Kyungseo Machine Co., Incheon, Korea). The extract solution was filtered using a standard sieve (No. 270, 53 *μ*m, Chung Gye Sang Gong Sa, Seoul, Korea), evaporated to dryness at 40°C under a vacuum (Eyela N-11, Tokyo, Japan), and freeze-dried (PVTFD10RS, IlShinBioBase, Yangju, Korea). The amount of water extract was 681.2 g (yield: 22.7%). Rats were administered with the ORS water extract of 100 mg/kg/day orally, equivalent to a single dose for an average human adult (28.1 g of the mixture of the dried herbs of ORS [21]). Two marker components, coumarin (0.37 ± 0.01 mg/g, *M* ± SD) and cinnamaldehyde (0.05 ± 0.00 mg/g, *M* ± SD), in ORS water extract were identified and quantified by the HPLC [21].

### 2.2. Animals

Age-matched male Sprague-Dawley rats (140–150 g, 5 weeks old, Samtako Inc., Osan, Korea) were used in the present experiments. Rats were maintained in an air-conditioned animal care room with 40–50% humidity at a 12–12 h light-dark cycle. Rats were housed individually in metabolic cages (Tecniplast, Buguggiate, Italy). Food (Cargillagripurina, Kunsan, Korea) and water (distilled water) were supplied *ad libitum*. All animal procedures for care and experiments were approved by the Institutional Animal Care and Utilization Committee for Medical Science of Wonkwang University (WKU16-85) and complied with the NIH *Guide for the Care and Use of Laboratory Animals*. Procedures for animal experiments adhered to the ARRIVE guidelines.

### 2.3. Goldblatt Model of Renovascular Hypertension

We used the Goldblatt model of renovascular hypertension. The left renal artery close to the abdominal aorta was clipped with a silver clip (0.2 mm slit) through the left flank incision under anesthesia with ketamine (25 mg/kg, i.m.) and xylazine (5 mg/kg, i.m.) as previously reported [22, 23]. Sham rats were prepared as the 2K1C except touching the left renal artery. Changes in systolic blood pressure (SBP) were traced by measurements weekly with tail-cuff plethysmography (MK-2000, Muromachi Kikai, Tokyo, Japan). Two-kidney, one-clip rats over 150 mmHg of systolic blood pressure were used as hypertensive rats. The levels of systolic blood pressure 150 mmHg are considered a stable and fully hypertensive range in this animal model of hypertension.

### 2.4. Protocols

Experiments were performed to test the effects of ORS on the renal function and systolic blood pressure in 4 groups of randomly selected rats (*n* = 34): Sham-operated rats treated with vehicle distilled water (Sham, *n* = 8); Sham rats treated with ORS (Sham-ORS, *n* = 8); 2K1C hypertensive rats treated with vehicle (2K1C, *n* = 9); 2K1C rats treated with ORS (2K1C-ORS, *n* = 9). Sample size was not different from the start to the end of the experiments. Administration of ORS (100 mg/kg/day orally in 1 ml of distilled water, an equivalent amount of a single dose of ORS in adults) or vehicle (distilled water in 1 ml orally) was continued for 3 weeks after 4 weeks of renal arterial clipping. Vehicle or ORS was administered between 5 : 00 and 6 : 00 p.m. Changes in systemic RAS and NPS in plasma were checked. Renin contents and gene expression of renin, vasopressin subtype 2 receptor (V2R), and aquaporin 2 (AQP2) were also measured in this series. In another series of experiments, the changes in the gene expression of AT_1_ receptor, Mas receptor, AT_2_ receptor, ACE, ACE2, and NHE3 were measured. Samples from Sham and hypertensive rats were analyzed simultaneously. SBP was measured once a week during the experimental period in a quiet and comfortable room. Urine samples for night (6 p.m. through 9 a.m. the next day) and day (9 a.m. through 5 p.m.) were collected separately [22]. Rats were killed with guillotine to avoid the influence of acute changes in atrial dynamics [24, 25] and anesthetics [26–28]. Blood was collected quickly in prechilled EDTA-coated or heparinized tubes. Plasma samples were separated and kept at −70°C until used. Tissue samples for gene expression were snap-frozen in liquid nitrogen and stored at −70°C until used.

### 2.5. Renal Function Test

All chemical assays were performed as reported previously [22]. Urine samples were collected two times in a day (9 : 00 a.m. and 6 : 00 p.m.) and centrifuged for 15 min, and the supernatants were kept in a refrigerator until used. Chemical assays were completed within 12 h. The concentration of electrolytes (Na^+^, K^+^, and Cl^−^) was measured using Electrolyte Analyzer (NOVA Biochemical, Waltham, MA, USA) and osmolality using Advanced Osmometer (Advanced Instruments Inc., Norwood, MA, USA). Creatinine concentration of urine and plasma was measured by a colorimetric method by Jaffe reaction using a spectrophotometer.

### 2.6. Radioimmunoassay for PRA, Aldosterone, and ANP

After guillotine, blood was collected quickly in prechilled EDTA-coated or heparinized tubes. Plasma samples were separated and kept at −70°C until used. Plasma and tissue levels of renin activity and plasma ANP were measured by radioimmunoassay as reported previously [22, 29]. Changes in renal renin contents were evaluated by angiotensin I (Ang I) generated with enough renin substrate from nephrectomized male rats and a small amount of whole kidney extract. Plasma renin activity was evaluated by Ang I generated with 1 ml of plasma. Radioimmunoassay for ANP was performed as reported previously [29]. The radioimmunoassay was performed in Tris-acetate buffer containing 0.2% neomycin, 1 mM EDTA, 50 BAEE-U/ml soybean-trypsin inhibitor, 0.02% sodium azide, 0.0004% phenylmethylsulfonyl fluoride, and 1% bovine serum albumin. Standards or samples were incubated following the addition of 100 *μ*l anti-ANP-antibody and 100 *μ*l [^125^I]-ANP for 24 h at 4°C. The separation of free tracer from antibody-bound tracer was conducted by adding 1 ml of dextran charcoal suspension. Plasma levels of aldosterone (ALDOCTK-2, DiaSorin Inc., Stillwater, MN, USA) were measured by radioimmunoassay as recommended by manufacturers.

### 2.7. RNA Isolation and Quantitative Real-Time PCR

RT-PCR was performed as reported previously [22, 23]. Total RNA of the kidney and heart tissues was extracted using TRIzol reagent (Ambion, Carlsbad, CA, USA) according to the manufacturer's instructions. The cDNA was synthesized using the High Capacity RNA-to-cDNA kit (Applied Biosystems, Waltham, MA, USA). The cDNA was used or immediately stored at −20°C. The sequences of primers were as shown in Table 1. The real-time PCR was performed by an initial denaturation step at 95°C for 10 min, followed by 40 cycles at 95°C for 15 sec, and finally 60°C for 60 sec in the Step-One Real-Time PCR system (4376600, Applied Biosystems, Foster City, CA, USA). Each RNA sample was measured in triplicate and normalized to the GAPDH. Data normalization of gene expression was conducted by StepOne™ software version 2.3 (Applied Biosystems, Waltham, MA, USA).

### 2.8. Reagents

Chemicals for buffer solution, including radioimmunoassay buffers for ANP and PRA radioimmunoassay, were purchased from Sigma-Aldrich, Yongin, Korea).

### 2.9. Statistical Analysis

The significance of the experimental results was validated by two-way ANOVA or repeated measures ANOVA with Bonferroni's multiple comparison test and Student's paired or unpaired *t*-test. Statistical significance between groups was accepted at the *P* < 0.05 levels. All results were presented as mean ± SEM. Statistical analysis was performed using SigmaPlot (version 10.0; SPSS Inc., Chicago, IL, USA) or GraphPad Prism 5.0 (GraphPad Software Inc., San Diego, CA, USA).

## 3. Results

### 3.1. ORS Enhanced Renal Function in Hypertensive Rats

**Figure 1:**
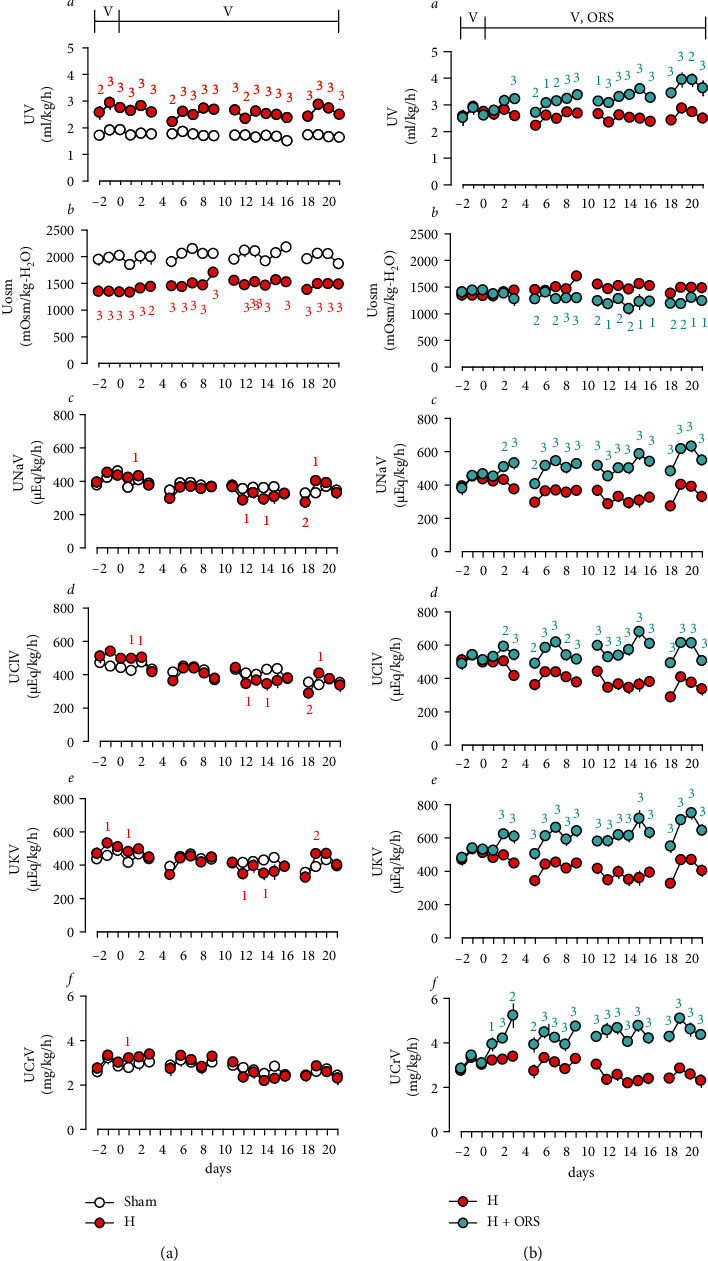
Changes in renal function in Sham-operated normotensive (Sham) and two-kidney, one-clip (2K1C) hypertensive rats treated with vehicle (H) (a) and H and 2K1C hypertensive rats treated with Oryeongsan (ORS) (*H* + ORS) (b). ○, Sham; 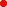, H; 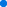, *H* + ORS. Vehicle, distilled water, 1 ml/rat/day, or ORS, 100 mg/kg/day orally, administered for 3 weeks after 4 weeks of operation. UV, urine volume; Uosm, urinary osmolality; UNaV, urinary excretion of Na^+^; UClV, urinary excretion of Cl^−^; UKV, urinary excretion of K^+^; UCrV, urinary excretion of creatinine. Number of experiments: Sham (Sham + vehicle), *n* = 8; H (2K1C + vehicle), *n* = 9; *H* + ORS, *n* = 8. Many of the standard error bars are embedded in the markings of the mean values. Small numerals above or below markings of the mean values are *P* values; 1, *P* < 0.05; 2, *P* < 0.01; 3, *P* < 0.001 versus Sham or H.

**Figure 2:**
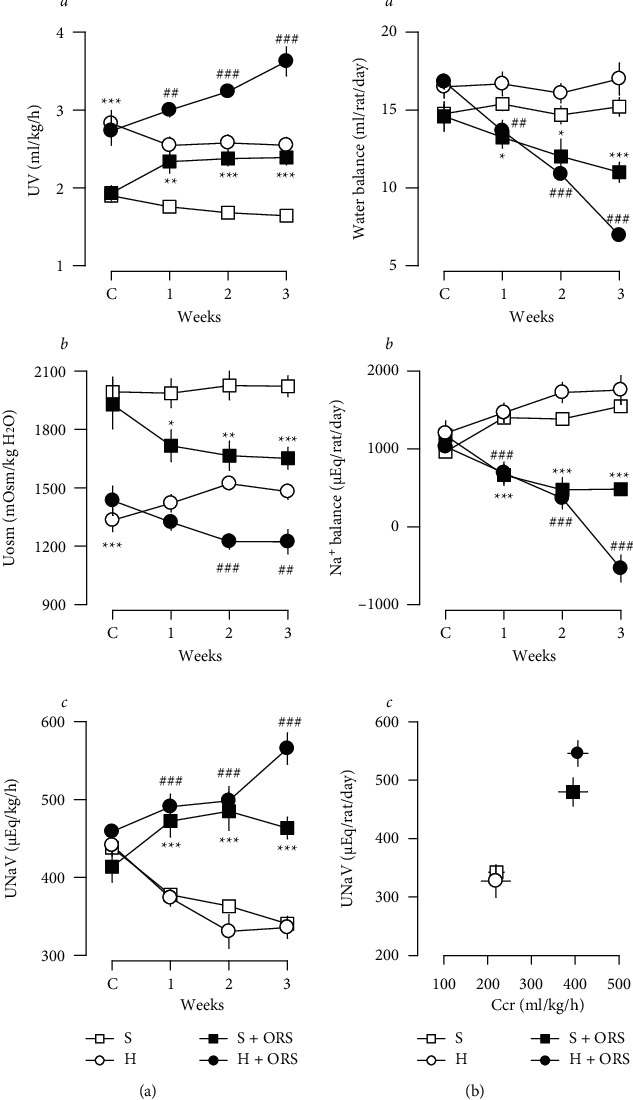
Summarized effects of Oryeongsan (ORS) on the urinary volume (UV), osmolality (Uosm), and Na^+^ excretion (UNaV) (a), water and Na^+^ balance (b), and the relation between glomerular filtration rate (GFR, C_Cr_) and U_Na_V (c) in Sham (S) and 2K1C hypertensive (H) rats treated with vehicle or ORS. C_Cr_, clearance for creatinine (GFR). Number of experiments: Sham + vehicle (S) or *S* + ORS, *n* = 8 for each group; *H* + vehicle (H), *n* = 9; *H* + ORS, *n* = 8. ^*∗*^*P* < 0.05, ^*∗∗*^*P* < 0.01, and ^*∗∗∗*^*P* < 0.001 versus Sham; ##*P* < 0.01 and ###*P* < 0.001 versus H. C, control values of 2 control days, day 1 and day 0; week 1, the first week of ORS administration, days 1–3 and 5–7 (6 days); week 2, the second week of ORS administration, days 8, 9, and 11–14 (6 days); week 3, the third week of ORS administration, days 15, 16, and 18–21 (6 days). Days 4, 10, and 17 were for blood pressure measurements and no urine collection.

**Figure 3:**
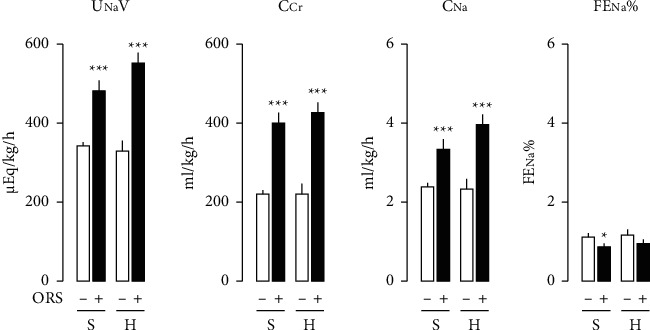
Changes in renal function in Sham and hypertensive rats treated with vehicle (open bars) or Oryeongsan (ORS) (closed bars). Effects of ORS on U_Na_V, C_Cr_, C_Na_, and FE_Na_% in Sham (S) and 2K1C hypertensive (H) rats. U_Na_V, urinary excretion of Na^+^; C_Cr_, clearance for creatinine (GFR); C_Na_, clearance for Na^+^; FE_Na_%, fractional excretion of Na^+^ in per cent. Number of experiments: Sham + vehicle (S-) or Sham + ORS (*S*+), *n* = 8 for each group; 2K1C + vehicle (H-), *n* = 9; or 2K1C + ORS (*H*+), *n* = 8. ^*∗*^*P* < 0.05 and ^*∗∗∗*^*P* < 0.001 versus corresponding control.

During the period of experiments, basal levels of urinary flow (UV) were stable in Sham and 2K1C hypertensive rats treated with vehicle (Figures 1(a) and 1(b)). Basal levels of UV and urinary osmolality (Uosm) were significantly different between Sham and hypertensive rats treated with vehicle, as higher in UV and lower in Uosm in hypertensive compared to normotensive Sham rats (Figure 1(a)). Basal levels of urinary excretion of Na^+^ (UNaV) and other electrolytes, including K^+^ and Cl^−^, were not significantly different between hypertensive and Sham rats treated with vehicle. ORS significantly increased UV, UNaV, urinary excretion of K^+^ (UKV), and Cl^−^ (UClV) and decreased Uosm in hypertensive as well as normotensive Sham rats (Figures 1(b)B and 2(a)B). ORS increased urinary excretion of creatinine (UCrV) and creatinine clearance (C_Cr_, glomerular filtration rate (GFR)) (Figures 1(b)F and 2(c)). The change of UNaV by ORS was a function of the GFR (Figure 2(c)). As shown in Figure 2(b), ORS elicited negative water (Figure 2(b)A) and Na^+^ balance (Figure 2(b)B), indicating a decrease in body water and Na^+^ accumulation.  Figure 3 depicts the change of renal function parameters in rats treated with ORS. ORS increased GFR (C_Cr_) and C_Na_, but not FE_Na_% changes.

### 3.2. ORS Downregulates Abundance of the AQP2 and V2R Expression in the Kidneys from Hypertensive Rats

**Figure 4:**
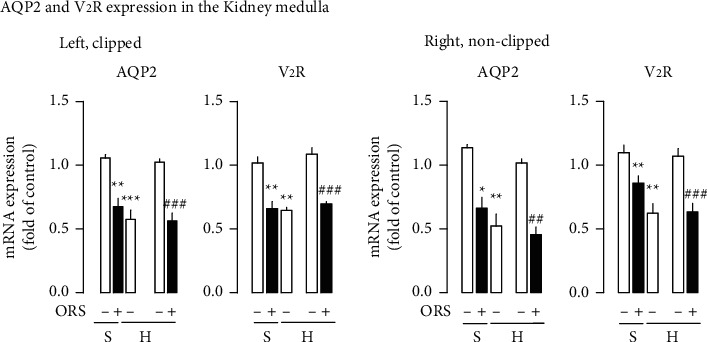
Effects of Oryeongsan (ORS) on the gene expression levels of vasopressin V_2_ receptor (V_2_R) and aquaporin AQP2 in the renal medulla from Sham (S) and hypertensive (H) rats. Open bar, vehicle-treated; closed bar, ORS-treated. Number of experiments: for gene expression of AQP2 and V2R (left and right kidneys), *n* = 3 for each of the Sham groups and *n* = 4 for each of the hypertensive groups. Sham + vehicle (S-) or Sham + ORS (*S*+); 2K1C + vehicle (H-); 2K1C + ORS (*H*+). ^*∗*^*P* < 0.05, ^*∗∗*^*P* < 0.01, and ^*∗∗∗*^*P* < 0.001 versus Sham; ##*P* < 0.01 and ###*P* < 0.001 versus H. In gene expression of the hypertensive group (H), there are 2 open bars in succession with different meanings: the first one is gene expression to be normalized compared to S- (Sham group treated with vehicle), and the other is to be normalized compared to H- (the hypertensive group treated with vehicle).

**Figure 5:**
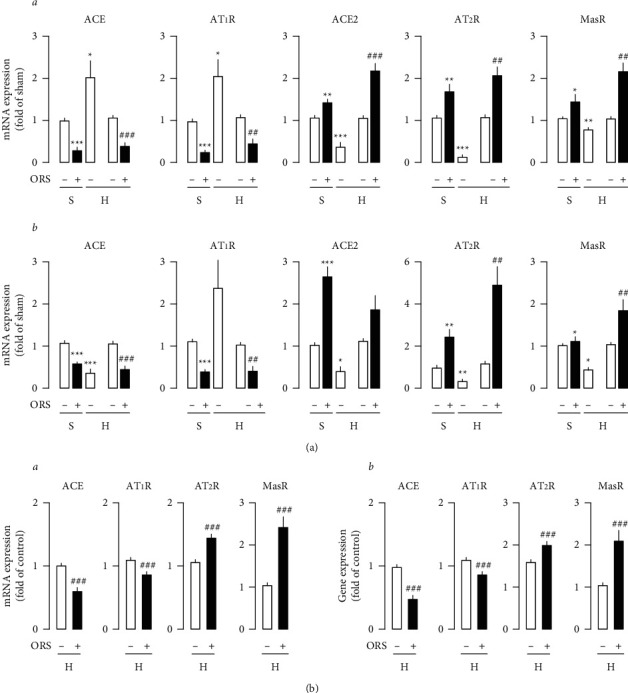
Effects of Oryeongsan (ORS) on the gene expression of the ACE, ACE2, AT_1_ receptor, AT_2_ receptor, and Mas receptor in the cortex of the kidneys from Sham (S) and hypertensive (H) rats (a) and ACE, AT1 receptor, AT2 receptor, and Mas receptor in the medulla of the kidneys from hypertensive rats (b). Number of experiments: *n* = 4 for mRNA expression of the ACE, AT1R, ACE2, AT2 receptor, and Mas receptor (a) in the left (A) and right (B) kidneys; *n* = 8-9 for mRNA expression of the ACE, AT1R, AT2 receptor, and Mas receptor in the left (A) and right (B) kidneys from hypertensive rats (b). Open bar, vehicle (-ORS); closed bar, ORS-treated (+ORS). In gene expression (a), there are 2 open bars in succession with different meanings in hypertensive rats treated with vehicle (H-): the first one is gene expression to be normalized compared to S- (Sham group treated with vehicle), and the other is to be normalized compared to H- (Hypertensive group treated with vehicle). ^*∗*^*P* < 0.05, ^*∗∗*^*P* < 0.01, and ^*∗∗∗*^*P* < 0.001 versus Sham (S-); ##*P* < 0.01 and ###*P* < 0.001 versus H-.

Clipping the renal artery decreased the AQP2 and V2R gene expression in the medulla of both kidneys from hypertensive rats (Figure 4). Treatment with ORS suppressed the abundance of the AQP2 and V2R gene expression in renal medulla from Sham and hypertensive rats (Figure 4). Furthermore, treatment with ORS suppressed the AT_1_ receptor and ACE gene expression and accentuated the AT_2_ receptor and Mas receptor expression in the renal medulla of both left and right kidneys from hypertensive rats (Figure 5(b)).

### 3.3. ORS Decreases Abundance of the Na^+^ Transporter NHE3 Expression Located in the Renal Cortex

**Figure 6:**
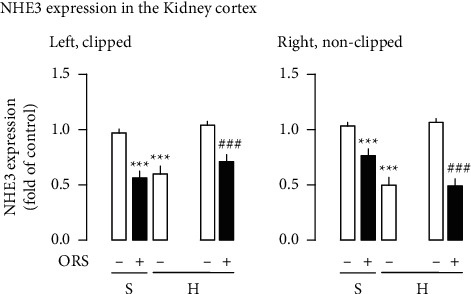
Effects of Oryeongsan (ORS) on the gene expression levels of NHE3 in the renal cortex from Sham (S) and hypertensive (H) rats treated with vehicle or ORS. Number of experiments: for left and right kidneys, *n* = 8 for each group. ^*∗∗∗*^*P* < 0.001 versus Sham (S-); ###*P* < 0.001 versus hypertensive rats treated with vehicle (H-); *H*+, hypertensive rats treated with ORS. In gene expression of the hypertensive group (H), there are 2 open bars in succession with different meanings: the first one is gene expression to be normalized compared to S- and the other is to be normalized compared to H-.

Figure 6 depicts modulation of the NHE3 expression. NHE3 expression was decreased in the cortex of the clipped and nonclipped kidneys from hypertensive rats. Treatment with ORS resulted in a decrease in the NHE3 expression. Similarly, ORS decreased NHE3 expression in the cortex of the left and right kidneys from Sham rats. Decrease of the NHE3 expression by ORS was accompanied by suppression of the ACE and AT_1_ receptor expression and accentuation of the ACE2, Mas receptor, and AT_2_ receptor expression in the cortex of the kidneys from Sham and hypertensive rats (Figure 5(a), A and B). ACE mRNA expression was increased in the left clipped kidney, while the expression decreased in the right nonclipped kidney.

### 3.4. ORS Modulates the Cardiorenal Hormone Systems, RAS and NPS, in Hypertensive Rats

**Figure 7:**
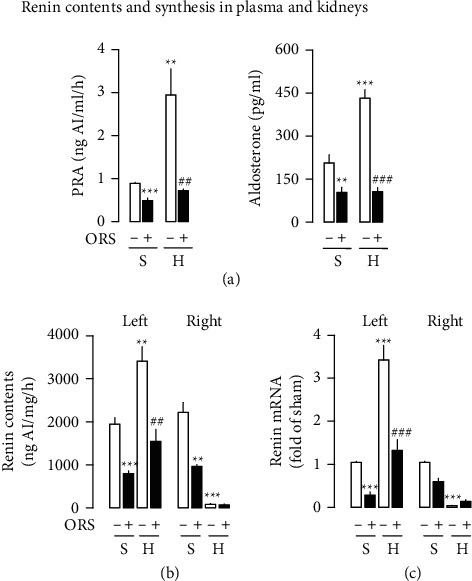
Effects of Oryeongsan (ORS) on the levels of plasma renin activity (PRA) and aldosterone (a), renin contents (b), and its gene expression (c) in the kidneys from Sham (S) and hypertensive (H) rats. Number of experiments: *n* = 8 for each of the Sham groups and *n* = 9 for each of the hypertensive groups for plasma levels of PRA and aldosterone; *n* = 4 for renin contents and renin mRNA of kidneys (left and right). Open bars, vehicle-treated (-ORS); closed bars, ORS-treated (+ORS). ^*∗∗*^*P* < 0.01 and ^*∗∗∗*^*P* < 0.001 versus Sham (S-); ##*P* < 0.01 and ###*P* < 0.001 versus hypertensive rats treated with vehicle (H-). (a) Plasma, (b) kidney, and (c) renin mRNA.

In hypertensive rats treated with vehicle, plasma levels of renin activity (PRA) and aldosterone were significantly elevated by about 233 and 109% compared to those of Sham rats, respectively (Figure 7(a)). Treatment with ORS decreased the systemic levels of components of the RAS by about 75–77% of the plasma levels of hypertensive rats treated with vehicle. Similarly, ORS decreased the plasma levels of PRA and aldosterone by about 45∼54% in Sham rats. To further define the modulation by ORS of the gene expression, components of the RAS in the kidney from hypertensive rats were dissected by molecular analyses. In hypertensive rats, renin contents and its synthesis were different between the clipped left and nonclipped right kidneys: accentuation in the clipped and suppression in the nonclipped (Figures 7(b) and 7(c)). Clipping the left renal artery modulated the expression of intrarenal components of the RAS differently in both kidneys from hypertensive rats. The levels of renin contents were increased by about 75% in the clipped left kidney from hypertensive rats treated with vehicle compared to the left kidney from Sham rats, while those were decreased by 96% in the unclipped right kidney (Figure 7(b)). Treatment with ORS decreased renin contents by about 55% in the left kidney. It was practically difficult to test the effects of ORS on the renin content in the right unclipped kidney because the levels were too low. Treatment with ORS decreased renin levels by about 59 and 57% in the left and right kidneys from Sham rats, respectively. Similarly, renin gene expression increased by about 229% in the left kidney from hypertensive rats treated with vehicle compared to Sham rats, while that was suppressed by about 97% in the right unclipped kidney from hypertensive rats treated with vehicle compared to Sham rats (Figure 7(c)). Treatment with ORS suppressed renin gene expression by about 61% in the left kidney from hypertensive rats compared to the levels in the left kidney from hypertensive rats treated with vehicle. Also, treatment with ORS suppressed renin gene expression by 73 and 43% in the left and right kidneys from Sham compared to the levels of the left and right kidneys from Sham rats treated with vehicle, respectively. These findings show that renin contents and renin gene expression were accentuated in the clipped left kidney compared to the kidney from Sham rats, while the levels were suppressed in the nonclipped right kidney from hypertensive rats treated with vehicle (Figures 7(b) and 7(c)).

Similarly, ACE gene expression was accentuated in the clipped left kidney from hypertensive rats compared to the kidney from Sham, while the levels were suppressed in the nonclipped right kidney (Figure 5(a), A and B). Treatment with ORS suppressed ACE gene expression by about 64% and 59% in the clipped and nonclipped kidneys from hypertensive rats, respectively (Figure 5(a), A and B). AT_1_ receptor expression significantly increased in both clipped and nonclipped kidneys, while ACE2, Mas receptor, and AT_2_ receptor expression decreased in both (Figure 5(a), A and B). Treatment with ORS reversed the accentuated ACE and AT1 receptor expression and the suppressed ACE2, Mas receptor, and AT_2_ receptor expression in the kidneys from hypertensive rats. ORS decreased abundance of the ACE and AT_1_ receptor expression, while it increased ACE2, Mas receptor, and AT_2_ receptor expression in the kidneys from Sham and hypertensive rats.

**Figure 8:**
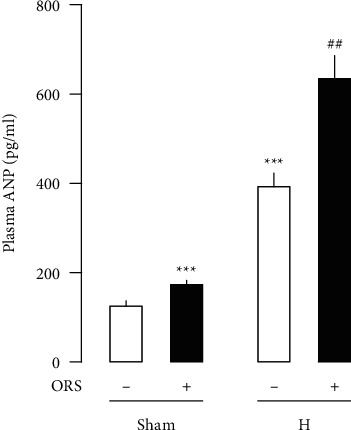
Effects of Oryeongsan (ORS) on the plasma levels of atrial natriuretic peptide (ANP) in Sham and hypertensive rats. Number of experiments; *n* = 8 for each group. -, rats treated with vehicle; +, rats treated with ORS. ^*∗∗∗*^*P* < 0.001 versus Sham treated with vehicle; ##*P* < 0.01 versus H-.

In hypertensive rats treated with vehicle, plasma levels of ANP significantly increased by about 226% compared to Sham rats treated with vehicle (Figure 8). Treatment with ORS further increased plasma levels of ANP by about 60% in hypertensive rats compared to hypertensive rats treated with vehicle. ORS further increased plasma levels of ANP in Sham rats by about 39%.

**Figure 9:**
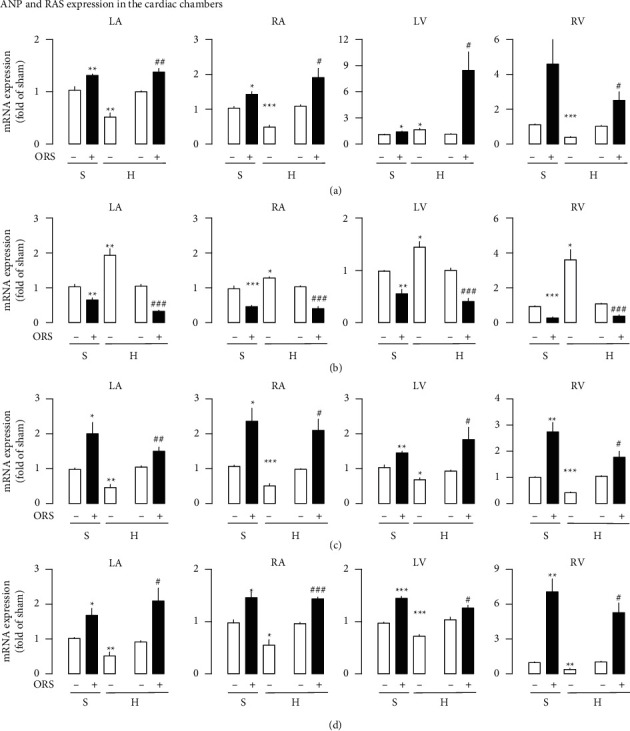
Effects of Oryeongsan (ORS) on the mRNA expression of the ANP (a), AT_1_ receptor (b), AT_2_ receptor (c), and Mas receptor (d) in the cardiac chambers from Sham (S) and hypertensive (H) rats. Number of experiments: *n* = 4 for each group of mRNA expression of ANP, AT_1_ receptor, AT_2_ receptor, and Mas receptor. Open bars, treated with vehicle (-ORS); closed bars, treated with ORS (+ORS). LA, left atrium; RA, right atrium; LV, left ventricle; RV, right ventricle. In mRNA expression, there are 2 open bars in succession with different meanings in H- group: the first one is gene expression to be normalized compared to S- (Sham group treated with vehicle), and the other is to be normalized compared to H- (hypertensive group treated with vehicle). ^*∗*^*P* < 0.05, ^*∗∗*^*P* < 0.01, and ^*∗∗∗*^*P* < 0.001 versus Sham (S-); #*P* < 0.05, ##*P* < 0.01, and ###*P* < 0.001 versus hypertensive group treated with vehicle (H-).

ANP gene expression was suppressed by about 56% in the cardiac chambers except left ventricle in hypertensive rats treated with vehicle compared to chambers from Sham rats (Figure 9(a)). ANP gene expression increased by about 51% in the left ventricle from hypertensive rats treated with vehicle. Treatment with ORS increased ANP gene expression by about 650% in the left ventricle and by about 86% in each of the remaining cardiac chambers from hypertensive rats. ORS increased ANP gene expression by about 103% from each of the cardiac chambers from Sham rats. Cardiac gene expression of the AT_1_ receptor was accentuated by 115% in each of the four cardiac chambers from hypertensive rats treated with vehicle, while that of the AT_2_ receptor and Mas receptor was suppressed by about 50% and 45%, respectively, compared to Sham rats treated with vehicle (Figures 9(b)–9(d)). Treatment with ORS suppressed AT_1_ gene expression by about 64% in each of the four cardiac chambers from hypertensive rats compared to hypertensive rats treated with vehicle (Figure 9(b)). ORS accentuated AT_2_ receptor and Mas receptor expression by about 81 and 155%, respectively, in the heart from hypertensive rats (Figures 9(c) and 9(d)). Treatment with ORS reversed the change of the RAS components observed in the hypertensive heart chambers. ORS increased ANP gene expression in the four cardiac chambers from Sham and hypertensive rats. ORS-induced increase in abundance of the ANP gene expression was associated with suppression of the AT_1_ receptor and accentuation of the AT_2_/Mas receptor expression.

### 3.5. ORS Lowers High Blood Pressure in Renovascular Hypertensive Rats

**Figure 10:**
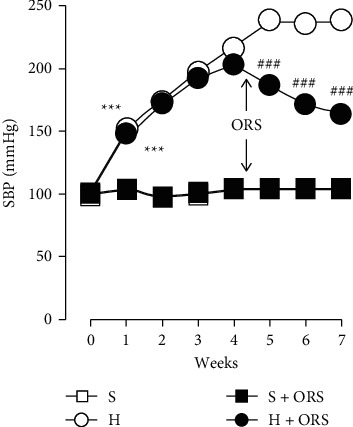
Time-dependent changes in systolic blood pressure (SBP) in Sham-operated normotensive (Sham) and hypertensive (H) rats treated with vehicle or Oryeongsan (ORS). Vehicle, 1 ml/rat/day, or ORS, 100 mg/kg/day orally, was administered for 3 weeks after 4 weeks of operation. Number of experiments: Sham + vehicle (S) or *S* + ORS, *n* = 8 for each group; hypertensive rats treated with vehicle (H-), *n* = 9; *H* + ORS, *n* = 8. ^*∗∗∗*^*P* < 0.001 versus Sham rats; ###*P* < 0.001 versus H. Arrows indicate the start of the treatment.

SBP showed no significant difference between groups before the operation, and renal arterial clipping significantly increased blood pressure after 1 week and gradually further increased in hypertensive rats (Figure 10). After 4 weeks of clipping the renal artery, ORS was administered for 3 weeks in Sham and hypertensive rats. Treatment with ORS significantly decreased SBP by 3.5 ± 1.3 (*P*=0.029), 11.6 ± 1.8 (*P* < 0.001), and 18.7 ± 2.2% (*P* < 0.001) of the pre-ORS levels (4th week, 199.5 ± 2.1 mmHg) after 1, 2, and 3 weeks, respectively, in the hypertensive group treated with ORS, while treatment with vehicle further increased SBP by 3.0 ± 2.0 (NS), 13.2 ± 3.7 (*P* < 0.01), and 18.8 ± 3.6% (*P* < 0.001) compared to the levels of SBP at 4th week (200.7 ± 3.0 mmHg) after 1, 2, and 3 weeks, respectively, in hypertensive rats treated with vehicle. In normotensive Sham rats, treatment with ORS or vehicle showed no significant effect on SBP and maintained stable levels of blood pressure.

## 4. Discussion

**Figure 11:**
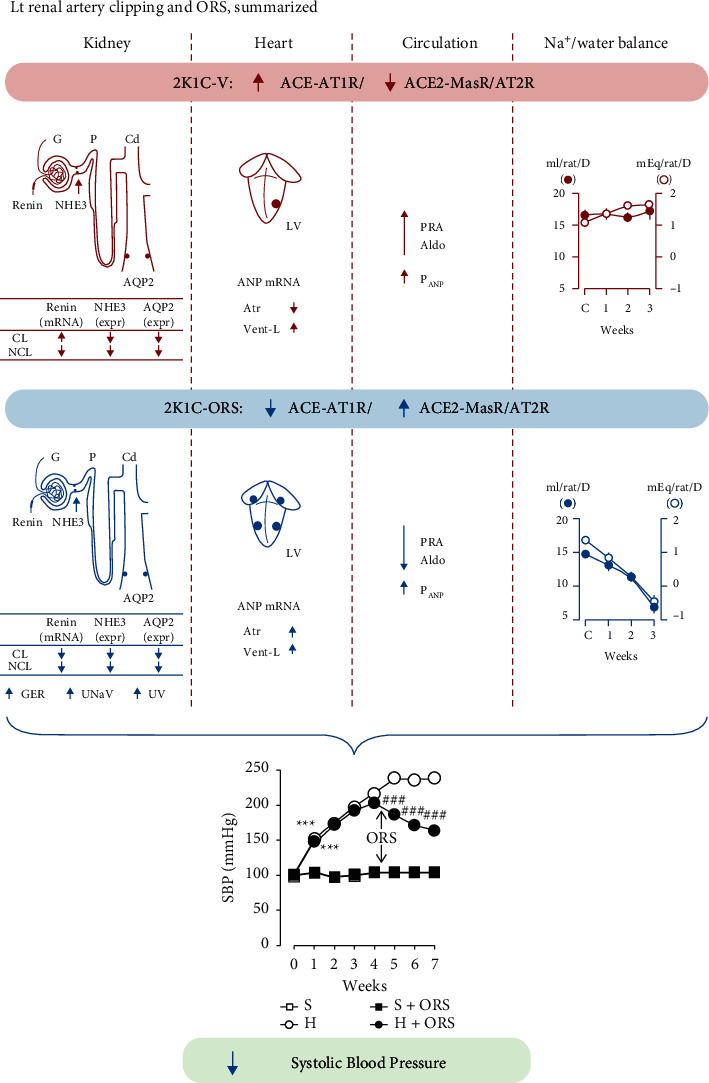
Summarized effects of Oryeongsan (ORS) and action mechanisms leading to amelioration of high blood pressure in 2K1C renovascular hypertensive rats. Clipping the left renal artery increased systolic blood pressure accompanied by accentuation of the systemic RAS and ACE-(Ang II)-AT_1_ receptor pathway and suppression of ACE2-(Ang-1-7)-Mas receptor/AT_2_ receptor signaling pathways in the kidney cortex and cardiac chambers. Cortical NHE3 and medullary AQP2 gene expressions were decreased. Renin synthesis was increased in the clipped kidney. Furthermore, ANP synthesis was accentuated in the left ventricle but suppressed in the atria. Plasma levels of PRA and aldosterone were increased. Treatment with ORS reversed the accentuated systemic RAS and ACE-AT_1_ receptor pathway and suppressed ACE2-Mas receptor/AT_2_ receptor signaling in the kidney cortex and cardiac chambers. Renin synthesis in the clipped kidney was decreased, and atrial ANP synthesis increased. Plasma levels of PRA and aldosterone decreased, and ANP increased. SBP decreased by ORS concomitantly with an increase in GFR, urinary excretion of Na^+^ and volume, and a decrease in body water and Na^+^ retention. All these processes induced by ORS were associated with a decrease in SBP. These findings lead us to conclude that chronic treatment with ORS, a medicinal herbal formula, elicits an antihypertensive effect through modulation of the cardiorenal function via modulation of the RAS and NPS. G, glomerulus; P, proximal renal tubule; Cd, collecting duct; NHE3, Na^+^-H^+^ exchanger 3; AQP2, aquaporin 2; 2K1C-V or H, 2K1C renovascular hypertensive rats treated with vehicle; 2K1C-ORS, 2K1C hypertensive rats treated with ORS; S, Sham-operated rats treated with vehicle; Sham + ORS, Sham-operated rats treated with ORS; CL, clipped kidney; NCL, nonclipped kidney; GFR, glomerular filtration rate; UNaV, urinary excretion of Na^+^; UV, urine volume; SBP, systolic blood pressure. C, control days; Atr, atria; Vent-L, left ventricle.

The present study shows that ORS lowers high blood pressure along with a decrease in body sodium (Na^+^) and water accumulation in hypertensive rats. These findings were accompanied by an increase in GFR with a decrease in abundance of the NHE3 expression in the renal cortex. Furthermore, ORS simultaneously modulated both the RAS and NPS expression. ORS suppressed the antinatriuretic and prohypertensive ACE-AT_1_ receptor axis and accentuated the natriuretic and antihypertensive ACE2-Mas receptor axis and AT_2_ receptor pathway in the cortex of the kidney (Figure 11). In addition, ORS modulated intracardial AT_1_ receptor, AT_2_ receptor, Mas receptor, and ANP gene expression. ORS decreased plasma levels of renin activity (PRA) and aldosterone and increased the levels of ANP, an endogenous counterregulator of the RAS. These findings indicate that ORS lowers high blood pressure through an increase in GFR with suppression of proximal tubular reabsorption, which leads to diuresis and natriuresis, resulting in a contraction of plasma volume and body salt accumulation in hypertensive rats. This is in relation to the reports that ablation of the AT_1_ receptor in the renal proximal tubule alone is sufficient to lower blood pressure in mice [13, 30]. All components of the RAS for ANG II generation and actions are present in the proximal convoluted tubules [11, 31]. Furthermore, the reports show that NHE3 expression is closely associated with the presence of the AT_1_ receptor [13, 14]. Ablation of AT_1_ receptor expression attenuated the ANG II-induced increase in NHE3 protein abundance [13]. The NHE3 located at the epithelial cells of the proximal tubules is the major site of the reabsorption of the glomerular filtrate [13, 15, 32]. Proximal tubular reabsorption through NHE3 is critically important in the regulation of blood pressure [13, 15]. In addition, AT_2_ receptor activation in the renal proximal tubules decreases blood pressure via the inactivation of NHE3 [33, 34]. AT_2_ receptor is a functional antagonist of the AT_1_ receptor in the regulation of blood pressure and Na ^+^ metabolism [33, 35]. AT_1_ receptor increases blood pressure via activation of NHE3 [13], whereas the AT_2_ receptor decreases via inactivation of NHE3 [33, 35]. In addition, the ACE2-ANG-(1–7)-Mas axis counteracts most of the deleterious actions mediated by the ACE-ANG II-AT_1_ receptor axis [34, 36, 37]. ANG II is a preferable substrate for ACE2 to produce ANG-(1–7) [38]. The present study shows that the ACE-AT_1_ receptor axis and ACE2-Mas axis/AT_2_ receptor pathway are inversely expressed in the kidney, clipped kidney in particular, from 2K1C hypertensive rats (Figure 11). Treatment with ORS reversed the expression pattern of these components. An increase in the ACE/ACE2 ratio may increase the ANG II levels in the kidney, whereas a decrease in the ratio may suppress the levels. Intrarenal increase of ANG II is associated with an increase in blood pressure [13, 39]. Proximal tubular reabsorption of the filtered Na^+^ via NHE3 activation is closely associated with the levels of ANG II in the kidney [13]. Therefore, the ORS-induced amelioration of high blood pressure is consistent with the notion that proximal tubular AT_1_ receptor, AT_2_ receptor, Mas receptor, and NHE3 are involved in the regulation of the body fluid and blood pressure homeostasis.

Although modulation of the RAS and NHE3 expression was similar to that of the 2K1C-ORS group, the changes in the blood pressure in the Sham-ORS group were not significant. The reason may be the different responses of the two groups in the Na^+^ and water balance to the treatment with ORS. The contraction of the Na^+^ and water balance of the Sham-ORS group is less than that of the 2K1C-ORS in the 3rd week in particular (Figure 2(b), A and B). This finding again indicates that the effect of ORS on blood pressure is closely associated with Na^+^ and water retention.

The present study shows that systemic and intrarenal RAS were modified in 2K1C hypertensive rats. Plasma levels of renin activity, ANG II, and aldosterone increased. Renal arterial clipping modified the intrarenal components of the RAS in the cortex. Renal renin contents and gene expression of renin and ACE were accentuated in the clipped kidney, whereas those were suppressed in the unclipped. AT_1_ receptor expression was upregulated in both kidneys from hypertensive rats, while the expression of ACE2, Mas receptor, and AT_2_ receptor was suppressed. Thus, clipping the renal artery accentuated the antinatriuretic and prohypertensive ACE-ANG II-AT_1_ receptor axis and suppressed the natriuretic and antihypertensive ACE2-ANG-(1–7)-Mas receptor axis/AT_2_ receptor pathway.

Vasopressin receptor, V2R, and AQP2 expressions were decreased in the renal medulla from hypertensive rats. Higher urinary flow and lower osmolality in the hypertensive rats compared to Sham rats may be associated with a decrease in abundance of the AQP2 and V2R expression. The changes in the systemic and intrarenal RAS observed in renovascular hypertension are consistent with the previous report [22]. Also, the suppressed expression of the AQP2 mRNA in the kidney from renovascular hypertension supports the previous report [40].

Treatment with ORS promoted renal function and ameliorated hypertension. These changes by ORS were associated with reversion of the systemic and intrarenal RAS: downregulation of the ACE-AT_1_ receptor axis and upregulation of the ACE2-Mas receptor axis/AT_2_ receptor pathway in hypertensive rats. The change of the NHE3 expression in the renal cortex was associated with the modulation of the RAS by ORS. Suppression of the systemic RAS by ORS may also be caused directly or indirectly through increased plasma levels of ANP [18, 19].

Treatment with ORS elicited diuresis and natriuresis in hypertensive (present results) as well as normotensive rats (present results and [2]). ORS-induced diuresis was accompanied by a decrease in urinary osmolality and downregulation of AQP2 and V_2_R expression in the renal medulla, in which downregulation of abundance of the ACE/AT_1_ receptor expression and upregulation of the Mas receptor/AT_2_ receptor expression were associated. This is in relation to the previous report [4]. Previously, Lee et al. showed that ORS suppressed AQP2 water channel expression and its apical membrane insertion in the renal medullary collecting duct cells [4]. It was also shown that ANG II-AT_1_ receptor signaling is involved in the regulation of the expression of AQP2 expression [41–43].

ORS increased urinary excretion of electrolytes, including Na^+^ (U_Na_V and C_Na_), concomitantly with an increase in GFR. The change by ORS of urinary excretion of Na^+^ was a function of GFR. ORS-induced natriuresis was not accompanied by an increase in the FE_Na_% changes but rather by a decrease in Sham rats. This shows the importance of the role of GFR in the ORS-induced changes in Na^+^ metabolism. These findings were coincident with a decrease in the NHE3 expression by ORS. This indicates that the ORS-induced increase in GFR with a decrease in the proximal tubular reabsorption of Na^+^ is closely associated with natriuresis and diuresis.

As for the changes in GFR, the present study shows that ORS increased GFR concomitantly with a decrease in the AT_1_ receptor expression and an increase in the AT_2_ receptor/Mas receptor in the renal cortex. Also, ORS suppressed systemic RAS and accentuated plasma levels of ANP. It is known that intrarenal blockade of the RAS with ACE inhibitor or ANG II receptor blocker increases GFR [44]. ANG II is an intrarenal RAS component to induce afferent glomerular arteriolar vasoconstriction. AT_1_ receptor is expressed in the vascular smooth muscle cells of both afferent and efferent arterioles [16, 31]. In addition, the role of the AT_2_ receptor expression is implicated in the increase in GFR. AT_2_ receptor is located in the afferent arterioles [34]. The present results are consistent with the reports. Furthermore, ANP is known to increase GFR through modulation of the glomerular afferent or efferent arterioles. ANP induces glomerular afferent arteriole vasodilation selectively [17, 45]. Also, it was shown that ANP dilates afferent and constricts efferent arterioles *in vitro* [46] and *in vivo* [47], which may increase glomerular capillary hydraulic pressure and glomerular plasma flow and lead to an increase in GFR.

Although the ANP synthesis of the cardiac chambers except the left ventricle was suppressed in 2K1C hypertensive rats, treatment with ORS significantly increased ANP gene expression, including both atria and plasma levels of ANP. Suppression of the AT_1_ receptor and accentuation of the AT_2_ receptor expression by ORS may be closely associated with the increase in the atrial release of ANP. Previously, it was shown that modulation of the intracardial expression of the AT_1_ receptor and AT_2_ receptor expression was associated with an accentuation of the secretion of ANP in the atria from hypertensive rats [23, 48, 49]. Furthermore, a recent study shows that chronic treatment with ORS accentuated the suppressed ANP secretion in the perfused beating atria from spontaneously hypertensive rats along with a decrease in abundance of the AT_1_ receptor and increase of AT_2_ receptor and Mas receptor expression [50]. It is worthwhile to comment on the role of accentuation of the atrial synthesis and release of ANP in the regulation of body sodium and water homeostasis in hypertension [51].

In a safety study of the ORS water extract, chronic toxicity was assessed in rats of both genders [20]. Rats were given ORS water extract by oral gavage at 0, 1000, 2000, and 5000 mg/kg/day for 4 weeks. The test showed no significant toxicological changes at the doses tested. Therefore, the no observed adverse effect level of the ORS water extract was more than 5000 mg/kg/day for rats of both genders. Furthermore, a previous report also showed no genotoxicity of the ORS water extract in three mutagenicity assays (up to 5000 *μ*g/plate in the Ames test with *Salmonella typhimurium* and *Escherichia coli* strains; up to 5000 *μ*g/ml in the *in vivo* mammalian chromosomal aberration test using Chinese hamster lung cells; 2000 mg/kg body weight in the *in vivo* micronucleus test using ICR mice bone marrow) recommended by the Korea Food and Drug Administration [21]. These results show that ORS water extract may be safe for human subjects in the regular doses and periods of ORS water extract administration.

In summary, chronic treatment with ORS lowered systolic blood pressure in hypertensive rats. ORS-induced contraction of the body fluid and salt accumulation. ORS increased GFR along with a decrease in the cortical NHE3 expression in the kidney. ORS decreased urine concentration and induced a decrease in V2R and AQP2 expression in the renal medulla from hypertensive rats. These findings were associated with suppression of the systemic RAS and modulation of the intrarenal renin activity and its gene expression with an increase in the plasma levels of ANP. Furthermore, ORS attenuated the antinatriuretic and prohypertensive ACE-AT_1_ receptor axis and accentuated the natriuretic and antihypertensive ACE2-Mas receptor axis/AT_2_ receptor pathway in the kidney. Furthermore, ORS increased cardiac synthesis and plasma levels of ANP, which is an endogenous counterregulator for the RAS. Altogether, the present study shows that ORS ameliorates high blood pressure through modulation of at least two signaling pathways of the cardiovascular and renal functions, RAS and NPS (Figure 11).

## 5. Conclusion

The present study indicates that chronic treatment with ORS ameliorates high blood pressure through contraction of body fluid and salt accumulation concomitantly with an increase in GFR with a decrease in abundance of the NHE3 expression in the renal cortex in hypertensive rats. These findings were closely associated with modulation of the systemic and intrarenal RAS and cardiac NPS. The results shown here lead us to conclude that ORS elicits antihypertensive effects through modulation of the RAS and NPS. The present study provides a biological basis of ORS for the treatment of impaired regulation of the body fluid and blood pressure homeostasis.

## Figures and Tables

**Table 1:** Primer sequences for RT-PCR.

Gene	Sense/antisense
Renin	S: 5′-AGG CAG TGA CCC TCA ACA TTA CCA G-3′, A: 5′-CCA GTA TGC ACA GGT CAT CGT TCC T-3′
AT1R	S: 5′-CTC AAG CCT GTC TAC GAA AAT GAG-3′, A: 5′-TAG ATC CTG AGG CAG GGT GAA T-3′
AT2R	S: 5′- GAA TCC CTG GCA AGC ATC TTA T-3′, A: 5′-ATG TTG GCA ATG AGG ATA GAC AAG-3′
MasR	S: 5′-GGA TGC CAG AAT TGA ACA CAG A-3′, A: 5′-CAC TGG CCC TCC TGA TGA A-3
ACE	S: 5′- GGG CAT TGA CCT AGA GAC TGA TG-3′, A: 5′-CTT GGG CTG TCC GGT CAT AC-3′
ACE2	S: 5′- ACC AAA GCA TTA AAG TGA GGA TAA G-3′, A: 5′-GTT GTT GGT CCA TTC ATA TGC ATT-3′
NHE3	S: 5′-CTG AGG AGG AAC CGA GCA-3′, A: 5′-AGG CCC AGA ACG ATG AGT AG-3
ANP	S: 5′-GAG GAG AAG ATG CCG GTA G-3′, A: 5′-CTA GAG AGG GAG CTA AGT G-3′
V2R	S: 5′-TGT GGC TCT GTT TCA AGT GC-3′, A: 5′-GCC AGG ATC ATG TAG GAG GA-3′
AQP2	S: 5′-TGG ATT CAT GGA GCA ACC G-3′, A: 5′-CCC TCT CCA TTG GTT TCT CTG TT-3
GAPDH	S: 5′-GTC GGT GTG AAC GGA TTT G-3′, A: 5′-CTT GCC GTG GGT AGA GTC AT-3′

## Data Availability

The data sets used and/or analyzed during the current study are available from the corresponding author upon reasonable request.

## Abbreviations

ACE: Angiotensin-converting enzyme
ACE2: Angiotensin-converting enzyme2
ALDO: Aldosterone
ANP: Atrial natriuretic peptide
AQP2: Aquaporin2
AT_1_ receptor: Angiotensin II subtype 1 receptor
AT_2_ receptor: Angiotensin II subtype 2 receptor
C_Cr_: Clearance for creatinine
GFR: Glomerular filtration rate
2K1C: Two-kidney, one-clip
MasR: Mas receptor
NHE3: Na^+^-H + -exchanger isoform3
NPS: Natriuretic peptide hormone system
ORS: Oryeongsan
PRA: Plasma renin activity
RAS: Renin-angiotensin system
SHR: Spontaneously hypertensive rats
UClV: Urinary excreted amount of Cl^−^
UKV: Urinary excreted amount of K^+^
U_Na_V: Urinary excreted amount of Na^+^
Uosm: Urinary osmolality
UV: Urinary flow (volume)
V2R: Vasopressin subtype 2 receptor.

## Additional Points

ORS lowered systolic blood pressure in hypertensive rats. ORS induced contraction of fluid and salt accumulation and increased GFR along with a decrease in cortical NHE3 expression in the kidney. ORS decreased urine concentration and induced a decrease in V2R and AQP2 expression in the renal medulla of hypertensive rats. ORS increased cardiac synthesis and plasma levels of ANP, which is an endogenous counterregulator for the RAS. ORS suppressed the antinatriuretic and prohypertensive ACE-AT1 receptor axis and accentuated the natriuretic and antihypertensive ACE2-Mas receptor axis/AT2 receptor pathway in the kidney.
